# Pulmonary vascular complications of chronic liver disease: Pathophysiology, imaging, and treatment

**DOI:** 10.4103/1817-1737.78412

**Published:** 2011

**Authors:** Ali Nawaz Khan, Hamdan Al-Jahdali, Khalid Abdullah, Klaus L. Irion, Quratulain Sabih, Alaa Gouda

**Affiliations:** *North Manchester General Hospital, Manchester, Pennine Acute Hospitals NHS Trust, UK*; 1 *Pulmonary Division, King Saud University for Health Sciences, King Fahad Hospital, King Abdulaziz Medical City, Riyadh, Saudi Arabia*; 2 *Department of Hepatobiliary, King Abdulaziz University, King Fahad Hospital, King Abdulaziz Medical City, Riyadh, Saudi Arabia*; 3 *Cardiothoracic Centre, Liverpool NHS Trust, The Royal Liverpool University Hospital UK, University of New Mexico, USA*; 4 *Department of Internal Medicine, Albuquerque, USA*

**Keywords:** Hepatopulmonary syndrome, portopulmonary hypertension, pulmonary arteriovenous shunts, Yttrium-90 microsphere embolization hepatocellular carcinoma

## Abstract

To review the pathogenesis of pulmonary vascular complications of liver disease, we discuss their clinical implications, and therapeutic considerations, with emphasis on potential reversibility of the hepatopulmonary syndrome after liver transplantation. In this review, we also discuss the role of imaging in pulmonary vascular complications associated with liver disease.

Our objective was to review current knowledge and advances made in understanding pulmonary vascular complications of liver disease between 2000 and 2010. The MEDLINE data base was explored through PubMed, and bibliographies of 229 papers were reviewed. The selected papers included case studies and series reporting results from patients with the vascular complication of chronic liver disease (CLD). We added our own radiological and clinical experience during this period. As the success of liver transplantation (OLT) is the only available definitive treatment for CLD and its success is dependent on the presence or absence of pulmonary vascular complications associated with CLD; our primary search only included studies emphasizing these findings. Of importance are the hepatopulmonary syndrome (HPS) and portopulmonary hypertension (PPH). HPS is defined as a triad of liver disease, increased alveolar-arterial gradient at breathing room air, and evidence of intrapulmonary vascular dilatations.[1] HPS is analogous to the hepatorenal syndrome[2] and independently worsens prognosis and influences cirrhotic patient’s management and scoring systems and accelerate the evaluation process for OLT. PPH is a combination of pulmonary arterial hypertension (PH) and portal hypertension (PoH). HPS causes hypoxemia due to pulmonary vasodilatation and arteriovenous shunting (AVS).[3] Quantification of AVS is an important consideration when considering patients for OLT and Yttrium-90 microsphere embolization for hepatocellular carcinoma (HCC). It is important to emphasize that the knowledge of the lung–liver relationship assists in forming a meaningful differential diagnosis and influences therapy. Understanding of HPS has helped further our knowledge of the interaction of the liver and the lung and to this end imaging plays a crucial role in the workup of patients for OLT.

## The Hepatopulmonary Syndrome

### Pathophysiology

The HPS is characterized by a defect in arterial oxygenation induced by pulmonary vascular dilatation and pulmonary AV shunting in the setting of liver disease. HPS can affect all ages and occasionally occur in noncirrhotic patients with PoH and may also been reported with in mild liver disease.[4] The exact mechanism of HPS is not known but it is postulated that it is the result of alteration in the production or clearance of chemical mediators causing intrapulmonary vascular vasodilatation (IPVD) and significant arteriovenous shunting (AVS) through the lungs. A major complication of IPVD is the ventilation–perfusion mismatch.[3] Hypoxia occurs as a result of inability of oxygen to diffuse through the markedly dilated lung capillaries. The capillaries are known to dilate to 15–500 μm (*n* = 8–15 mm) in HPS.[5] Imbalance in the endothelin receptor response, pulmonary microvascular remodelling, and genetic predisposition is thought to lead to IPVD.[6]

HPS has three major components, i.e. liver disease, IVPD, and a defect in oxygenation. Increased alveolar-arterial gradient occurs in HPS while breathing room air.[3] The major defect that underlies HPS is hypoxemia secondary to IPVD associated with AVS. AVS occurs through the dilated pulmonary capillaries.[5] Pleural spider nivae also contribute to AVS.[1] Capillary vasodilatation is most pronounced at the lung bases; thus, explaining orthodeoxia and platypnea associated with HPS (see below). Pulmonary vasodilatation leads to increased pulmonary blood flow and an increase in cardiac output; an event that causes elevated perfusion ventilation mismatch (VQ mismatch) and AV shunts.[7,8] VQ mismatch appears to be a major event in the pathogenesis of hypoxemia in HPS as a result of extensive pulmonary vasodilatation, a decrease in V/Q ratio in alveolar-capillary units and resultant low PO_2_ and O_2_ content of blood leaving the lungs. Air trapping and poor ventilation in dependent parts of the lungs may present clinically as closing volume exceeding functional residual capacity. Decreased ventilation from air trapping leads to further V/Q mismatched in the dependent parts of the lungs. Lowered V/Q ratio results in elevated Δ (A-a) O_2_ correctable only by 100% oxygen inhalation. However hypoxemia caused by larger AV shunts does not respond to inhalation of 100% oxygen. A significant proportion of patients with HPS and advanced liver disease show diffusion impairment associated with decreased DLCO without concomitant increase of Δ (A-a) O_2_. This phenomenon explains the higher sensitivity of DLCO over Δ (A-a) O_2_ in the detection of pulmonary vasodilatation.[9–13]

Due to pulmonary capillaries dilatation oxygen encounters difficulty in diffusing into the centre of the larger capillaries. Increased cardiac output and the associated reduced transition time of blood through the pulmonary vascular bed also impair diffusion, leading to a diffusion–perfusion defect or alveolar capillary oxygen disequilibrium.[11]

Early investigators attributed IVPD in HPS to inadequate synthesis or metabolism of pulmonary vasoactive substances by a failing liver.[1,7] However, it has been shown that nitric oxide, a potent vasodilator, is exhaled in larger quantities in patients with cirrhoses related HPS and normalizes following OLT.[8] A single case report supports this theory where NG-LNAME, a nitric oxide production inhibitor, transiently improved HPS.[14] Similarly intravenous methylene blue given to patients with HPS experienced improvements in gas exchange accompanied by an increase in pulmonary vascular resistance.

Gram-negative intestinal endotoxemia in PoH associated with increased nitric oxide release and tumor necrosis factor have been proposed as a cause of excessive pulmonary vasodilatation.[15] Improvement in arterial oxygenation has been reported following antibiotic therapy.[16]

A recent study has thrown doubt on vasodilatation being the sole cause of the pathophysiology underlying HPS. It has been shown that administration of inhaled l-NAME in HPS patients did not improve V/Q mismatch and arterial hypoxemia.[17] This study suggests that vasodilatation alone is unlikely to explain the more than 10-fold increase in capillary diameters in HPS especially as there is little smooth muscle to relax in normal capillaries.

### Clinical presentation

Typically HPS presents with dyspnea of insidious onset, platypnea and orthodeoxia.[18] Physical examination may reveal dyspnea associated with cyanosis in 90% of all cases. The presence of clubbing has the highest positive predictive value (75%) and dyspnea the highest negative predictive value (100%) for HPS. Spider nevi are a common clinical feature of patients with HPS with significant relationship between cutaneous spider angiomata and systemic and pulmonary vasodilatation suggesting that spider nevi may represent a cutaneous marker for intrapulmonary vascular dilatations.[19,20]

Two types HPS have been described: Type I lesions are associated with vascular dilatations at the precapillary level close to the normal gas exchange units of the lung and Type II with focal larger dilatations amounting to AVS distant from the gas exchange units. Supplementary oxygen improves Type I HPS PaO_2_ but not Type 2 HPS.[1]

### Prognosis

HPS is associated with an increase risk of death, worse functional status, and quality of life in patients evaluated for OLT.[21] The median survival time in cirrhotic patients with HPS has been reported as 10.6 months compared to 40.8 months in cirrhotic patients without HPS. The leading cause of death is hemorrhagic shock secondary to gastrointestinal bleeding. The degree of arterial hypoxemia affects survival. Survival is worse with lower baseline PaO_2_(≤50 mmHg).[22]

### Diagnosis

Diagnosis is usually based on imaging and arterial gas analysis. Arterial gases are analysed initially to identify elevated alveolar-arterial differences in O_2_or hypoxemia in suspected HPS. There is controversy in the use of cut-off values of PaO_2_.[23] A variety of cut-off points have been used resulting in a wide range of prevalence of HPS. Schenk and colleagues suggested that arterial hypoxemia defined as a PaO_2_ <70 mmHg or below the age-related threshold predicted the presence of HPS with high probability in the absence of intrinsic cardiopulmonary diseases.[23] If PaO_2_ levels are abnormal a chest radiograph (CXR) and pulmonary function tests are used to help exclude other cardiopulmonary abnormalities. However, the demonstration of AV shunting despite the presence of other cardiopulmonary abnormalities by radiological means can achieve the diagnosis of HPS. In a cirrhotic patient suspected of HPS, it is important that other cardiopulmonary causes of hypoxia be excluded such as pulmonary atelectasis, ascites, chronic obstructive pulmonary disease, and hepatic hydrothorax.

A definitive diagnosis of HPS can be made by demonstration of pulmonary vasodilatation associated with functional arteriovenous shunting. Imaging studies that can identify such shunts include contrast echocardiography and perfusion scintigraphy with 99mTc, which are usually carried out following analysis of arterial gases to identify elevated alveolar-arterial differences in O_2_ or hypoxemia.

In the setting of HPS administration of 100% oxygen can be used as a noninvasive test to quantify pulmonary AVS. This is an important parameter to measure as demonstration of AVS suggests that the hypoxemia may not resolve following OLT. In response to breathing 100% oxygen if the PO_2_ levels rise to ≥600 mmHg, the presence of AVS is unlikely, but if levels fail to exceed 500 mmHg, the presence of a shunt cannot be ruled out. If PO_2_ levels fail to rise to above 150–200 μmHg, shunt is most probably the main mechanism of hypoxemia.[10]

**Figure 1:**
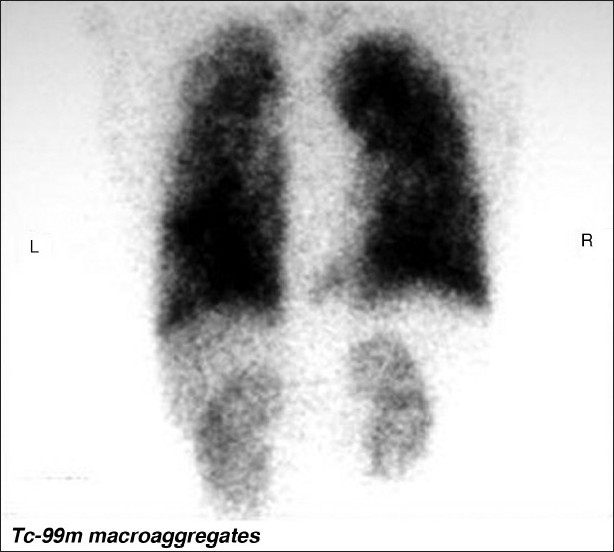
99Technetium macroaggregated albumin perfusion imaging in a patient with HPS shows a right to left shunt. The 99mTc-MMA has entered the systemic circulation and has perfused the kidneys

**Figure 2:**
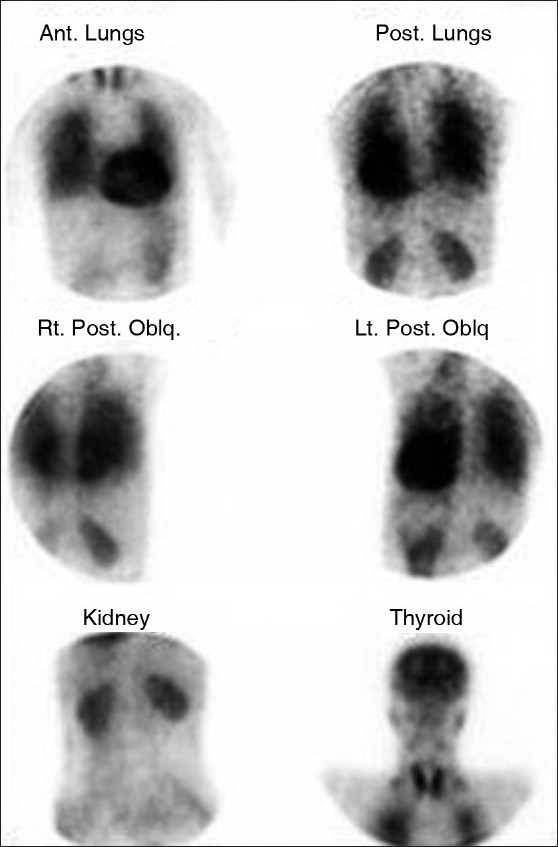
99Technetium macroaggregated albumin perfusion imaging in a patient with HPS shows a right to left shunt. The 99mTc-MMA has entered the systemic circulation and distributed to systemic organs. Normally, <5% of the isotope can be quantitated over the brain. In HPS, there is >6% uptake in the brain as in this case. (Courtesy Durre-e-Sabih Multan)

**Figure 3:**
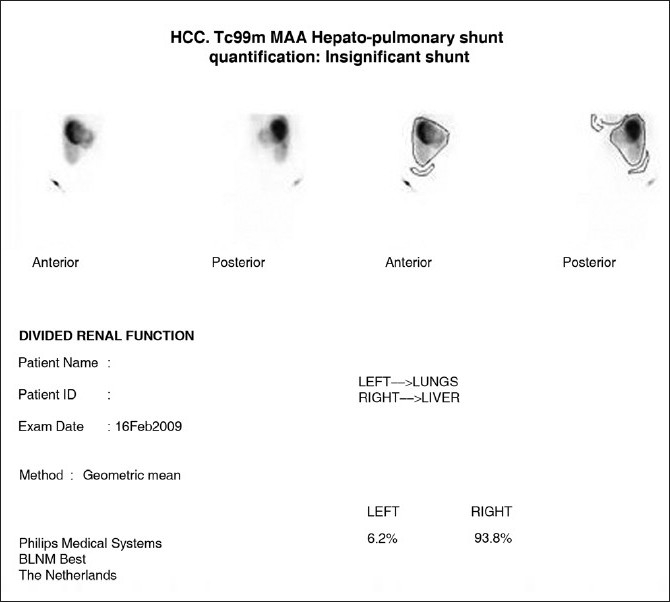
In preparation for hepatic radioembolization with Yttrium-90 glass microspheres 99Technetium macroaggregated is given via the hepatic artery and regions of interest are drawn over lungs, liver and background. Less than 5% systemic shunt of 99Technetium macroaggregated is normal. The 5–15% is insignificant and we proceed to Yttrium-90 glass microspheres with a standard 150 Gy dose. With a systemic shunt of 10–25%, the dose of Yttrium-90 glass microspheres is modified to 110-130 Gys. (Courtesy Dr. Ghulam Mustafa Shah, Department of Medical King Abdul Aziz Medical City Riyadh)

**Figure 4:**
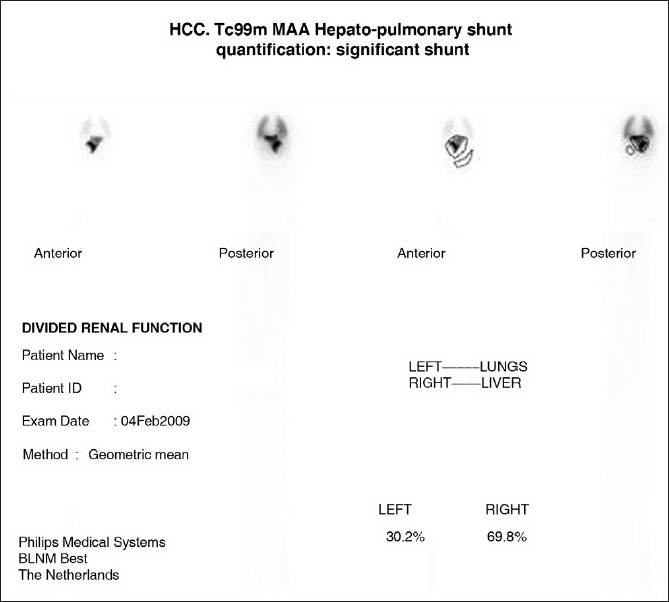
In preparation for hepatic radioembolization with Yttrium-90 glass microspheres 99Technetium macroaggregated is given via the hepatic artery and regions of interest are drawn over lungs, liver, and background. With a systemic shunt greater than 25% Yttrium-90 glass microspheres embolization is contraindicated. (Courtesy Dr. Ghulam Mustafa Shah Syed, Head Nuclear Medicine Section, Department of Medical Imaging, King Fahad Hospital, King Abdul Aziz Medical City Riyadh)

The presence of a ventilation–perfusion mismatch can be achieved by several means. The two methods available are ^99^Technetium macroaggregated albumin (99mTc-MMA) perfusion imaging or contrast echocardiography (CEE).[24] 99mTc-MMA perfusion scanning is a more sensitive procedure as it allows quantification of the degree of intrapulmonary shunting. 99mTc-MMA >20 mm in diameter are entrapped in pulmonary vasculature and undergo decay.[25] In patients with right to left shunt, the 99mTc-MMA enters the systemic circulation and distributed to systemic organs. Normally, less than 5% of the isotope can be quantitated over the brain. In HPS, there is >6% uptake in the brain. A disadvantage of 99mTc-MMA is that it is unable to differentiate intracardiac from intrapulmonary shunt [Figures 1–4]. As to which imaging modality is used depends on local availability both in terms of expertise and equipment. Contrast echocardiography is very much operator dependent and is more labor intensive. Most centres including our own prefer to use 99mTc-MMA perfusion scanning.

CEE is usually performed using intravenous microbubbles. These microbubbles are trapped in the pulmonary vasculature and absorbed. However in the presence of intracardiac right to left shunt, the microbubbles are seen in the left heart within the first three cardiac cycles.[26] In HPS, the microbubbles are usually seen in the left heart after the third heart beat.[26] Other findings that support the diagnosis of HPS include a maximal left atrial volume ≥50 ml in a cirrhotic patient. Right ventricular diastolic dysfunction is more common in cirrhotic patients with HPS than cirrhotic patients without HPS.[27] Transesophageal echocardiography using gelatin contrast is said to be more sensitive than transthoracic echocardiography for intrapulmonary shunt detection.[28] CEE fails to quantify the degree of intrapulmonary shunting and is unable to differentiate between intrapulmonary vascular dilatation and direct arteriovenous communication. A further limitation of CEE is lack of specificity as a positive study may occur in cirrhotic patients with a normal arterial blood gas profiles and thus by definition no HPS.

Pulmonary angiography (PA) is an invasive procedure, which uses iodinated contrast media and is usually reserved in patients with an increase in the PaO_2_ to less than 300 mmHg. However, presently PA is rarely performed in the diagnosis of HPS but has a role when CTA suggests an AVM when PA may be used when the AVM appears amenable to embolization. PA is also able to differentiate between Type I and II HPS.[29] Type I HPS is depicted as normal to advanced diffuse ‘spider-like’ or spongy appearance.[30]

**Figure 5:**
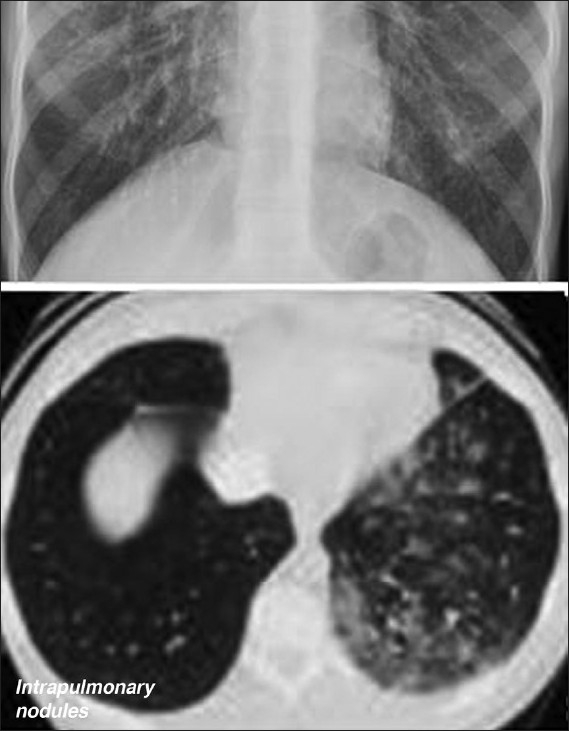
A coned view of a CXR of lung basis with HPS shows bibasilar nodular or reticulonodular opacities. These are seen in 5–13.8% of patients with CLD and 46–100% of patients with HPS. These opacities are shown to represent dilated lung vessels on computed tomography as shown

**Figure 6:**
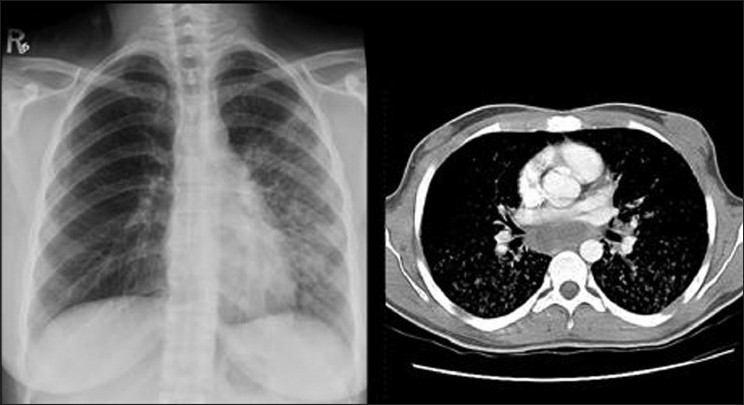
A CXR of lung basis of 47-year-old woman with HPS shows basilar reticulonodular opacities at the left lung base and left mid-zone. These opacities which represent dilated lung vessels are seen on both sides on CT as shown

**Figure 7:**
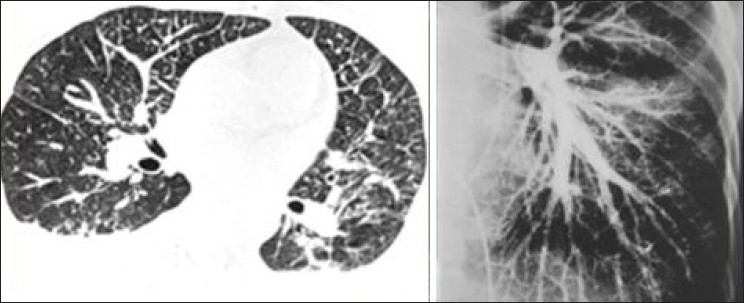
Axial contrast enhanced CT (right) and a pulmonary angiogram (left) show dilated pulmonary vasculature in a 53-year-old man with CLD associated with HPS

Most patients being cared for in hospital setting would have undergone a conventional CXR, which is not only useful in the diagnosis of HPS but is also crucial for excluding other causes of hypoxemia. A CXR in HPS shows bibasilar nodular or reticulonodular opacities in 5–13.8% of patients with CLD and 46–100% of patients with HPS.[30] These opacities are shown to represent dilated lung vessels on standard computed tomography [Figures 5–7]. High-resolution CT is useful in excluding pulmonary fibrosis or emphysema as the cause of these opacities. CT can identify decreased bronchoarterial ratio, which is highly specific for HPS.[25] However, a multimodality approach is necessary to depict cases of liver origin. Suga and associates used breath-hold pulmonary perfusion SPECT-CT images to characterize CT manifestations of intrapulmonary arteriovenous communications causing right-to-left shunt in two patients with HPS.[31] In both patients, whole body scans depicted systemic organs thus confirming the existence of right-to-left shunt. Breath-hold SPECT-CT fusion images show that perfusion defects are predominantly located at subpleural reticulo-nodular opacities and/or dilated vessels in the lung base.[31]

To summarise: the diagnostic flow chart of HPS generally starts with (1) arterial blood gas analysis performed in the supine position, (2) a CXR follows to rule out causes of hypoxemia other than HPS, (3) pulmonary function tests to include spirometry and DLCO. Spirometry is generally normal but may show a mild restrictive pattern due to pleural effusion or ascites. DLCO depicts decreased diffusion ability of lungs due to intrapulmonary vasodilatation,[32] (4) 99mTc-MMA perfusion scanning (CEE if local expertise is available), (5) CT is reserved for patients when pulmonary AVM is suspected as the cause of AV shunt, but may be performed to look at the liver when the chest can be included into the protocol, (6) pulmonary angiogram is carried out as a prelude to vascular intervention when CT angiography has demonstrated a pulmonary AVM.

### Therapeutic measures

No effective medical treatment is available, but better knowledge of the pathophysiology should improve this situation in the future. Medical management, transjugular intrahepatic portosystemic shunts (TIPS), and pulmonary arterial coil embolization have been described as temporizing measures until OLT is performed.[33]

Several therapeutic trials in HPS have shown poor results such as somatostatin analogues, cyclooxygenase inhibitors, and immunosuppressive agents such as corticosteroids and cyclophosphamide.[34] Almitrine bismesylate a selective pulmonary vasoconstrictor has achieved anecdotal success.[35] Inhaled prostanoid iloprost could improve quality of life in patients with HPS waiting for OLT and post-surgery until the resolution of the hypoxemia.[36] A small study using garlic (*Allium sativum*) powder capsules daily for a minimum of 6 months resulted in a modest improvement in arterial oxygenation in 6 out of 15 patients with HPS.[37] PoH appears to be a key component in development of HPS as a reduction in portal pressure appears to be beneficial in HPS.[38] Some reports have shown improvement in gas exchange with the use of TIPS in HPS.[38] Another study of TIPS did not show a sustained change in arterial blood gas profile.[39] Suprahepatic inferior vena cava obstruction in association with HPS responds to percutaneous balloon cavaloplasty experiencing intrapulmonary shunt reversal.[40]

OLT provides the only definitive treatment of HPS. Over 85% of patients with HPS undergoing OLT show complete reversal of HPS or experience a significant improvement in hypoxemia. This reversal may take up to year. However, patients with HPS undergoing OLT have a significant mortality of 71% at 1 year.[41]

**Figure 8:**
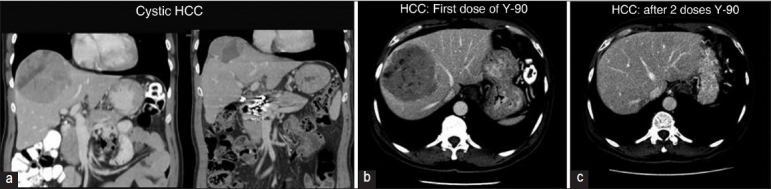
(a) Illustrate the application of hepatic radioembolization with Yttrium-90 glass microspheres 99Technetium following quantitative estimation of a right to left intracardiac shunt by a 99Technetium macroaggregated perfusion lung scan. These series of images (b, c) illustrate the benefit of hepatic radioembolization with Yttrium-90 glass microspheres in a cystic hepatocellular carcinoma. There is an initial necrosis of the tumor followed by almost complete shrinkage. (Courtesy Dr Ghulam Mustafa Shah Syed, Department of Medical Imaging, Abdul Aziz Medical City Riyadh)

**Figure 9:**
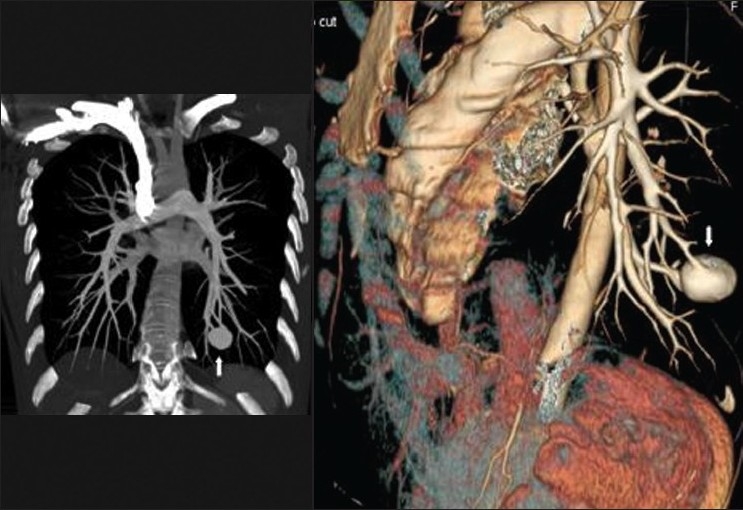
It is important that larger right to left shunts through pulmonary arteriovenous malformations are excluded by cross-sectional imaging when testing for HPS and when consideration is being given for hepatic radioembolization with Yttrium-90 glass microspheres. These coronal and sagittal reconstructions of contrast enhanced CT show a large arteriovenous malformation at the left lung base

Hepatic radioembolization with Yttrium-90 glass microspheres for treatment of inoperable primary liver cancer was first reported by Cao *et al*. It is presently considered a worthwhile therapeutic approach because of encouraging rate of response or stabilization. Initial work up includes assessment of the degree of AV shunt[42] [Figures 1–4, 8]. It is important that bigger intrapulmonary shunts are excluded by cross-sectional imaging. The same statement applies when dealing with HPS [Figure 9].

## Portopulmonary Hypertension

### Pathophysiology

PPH is defined by criteria obtained by right heart catheterization in patients with PoH and include: Elevated mean pulmonary artery pressure (>25 mmHg at rest, >30 mm Hg with exercise); increased pulmonary vascular resistance (>240 dynes s/cm^5^; and normal pulmonary artery occlusion pressure (<15 mmHg) or an elevated transpulmonary gradient.[43] The risk of developing PH increases with the duration of PoH without any clear relation to the degree of PoH, hepatic failure, or amount of blood shunted.[44] PH occurs in approximately 2% of patients with PoH due to either cirrhosis or extra hepatic lesions. Primary PH has been reported with extrahepatic PoH, portacaval shunt, and noncirrhotic portal fibrosis.[45]

**Figure 10:**
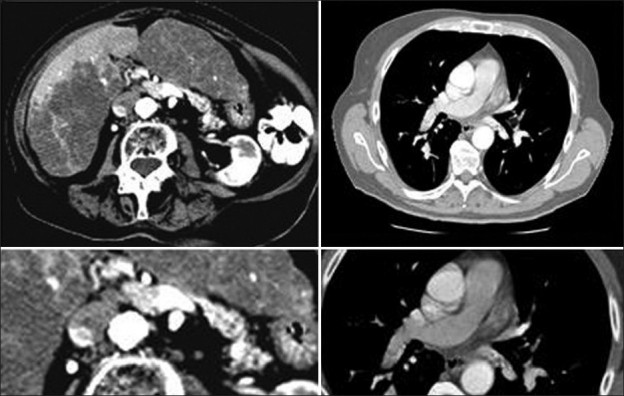
Axial contrast enhanced images of the liver and lungs on an 82-year-old woman with PPH showing portal vein thrombosis, which is associated with pulmonary embolism supporting the theory that PPH may be secondary to embolic material entering the lungs via the portosystemic venous shunts associated with PoH although no varices are seen in this patient

The cause of PPH is not well understood but may represent a combination of embolic and plexogenic factors. It is postulated that embolic material enters the lungs via portosystemic venous shunts associated with PoH [Figure 10]. Thromboembolic PH is a complication of congenital portosystemic venous shunt, and may be latently present in patients with PH of unknown etiology.[46] Plexogenic PH may develop as a result of plexogenic arteriopathy due to vasoactive agents. Vasoactive substances bypass the liver via the portosystemic shunts or are ineffectively cleared by the compromised liver. Endothelial damage with vascular remodeling due to excessive pulmonary blood flow and smooth muscle proliferation adds further to vasoconstriction.[47] Genetic variation in estrogen signalling and cell growth regulators is associated with the risk of PPH, which may explain the mechanism for its development in certain patients with severe liver disease.[48] PoH either precedes or is diagnosed concurrently with PH, supporting the hypothesis that in PH, the pulmonary circulation may be exposed to vasoactive substances not metabolized or produced by the cirrhotic liver, which possibly induce vasoconstriction or direct toxic damage to the pulmonary arteries.[49]

### Clinical presentation

Early stage of PPH is generally asymptomatic or patients may have symptoms of the underlying liver disease. It is therefore imperative to have a high index of suspicion and screen for PH. With advancing disease exertional dyspnea is the most frequent presenting symptom of PPH (81%); other symptoms, such as syncope, chest pain, and fatigue, are seen in a third of the patients. Auscultation may reveal an accentuated pulmonary component of the second heart sound and a systolic murmur. Hemodynamic findings included severe pulmonary hypertension with normal pulmonary capillary wedge pressure and cardiac output.[50]

### Prognosis

Patients with PPH carry high perioperative mortality and have a mean survival of 15 months.[50] As PPH increases perioperative death, screening for PPH is mandatory in patients undergoing evaluation for OLT.

### Diagnosis

Lung function tests in PPH are not generally helpful although diffusing capacity may be reduced. B-type natriuretic peptide testing is helpful in monitoring the severity of disease and the efficacy of treatment in PH; but its role in PPH has not been defined. EKG findings in PPH include right atrial or ventricular enlargement and a right bundle branch pattern. A CXR may show prominent pulmonary arteries and cardiomegaly in advance PH. Standard echocardiography is an excellent tool to detect signs of PH.[51] Findings suggestive of PPH include elevation of right ventricular systolic pressure (RVSP). Patients with an estimated RVSP greater than 50 mmHg undergo right heart catheterization.

Right heart catheterization (RHC) is regarded as a definitive test for confirmation of PPH, which allows accurate measurement of pulmonary artery pressures, pulmonary artery occlusion pressure, cardiac output, and pulmonary vascular resistance. RHC has the advantage of excluding other treatable causes of PH.

**Figure 11:**
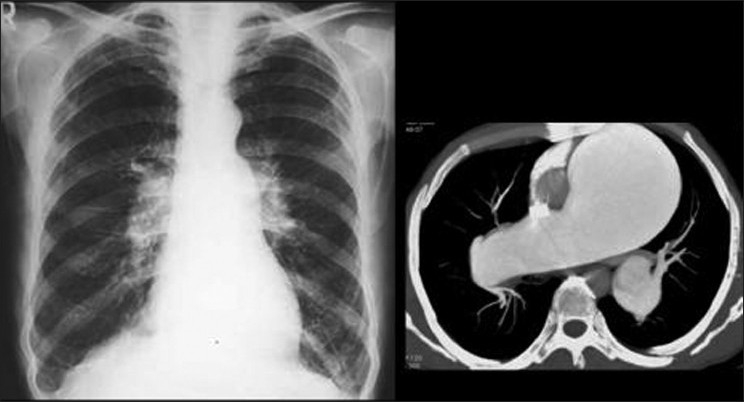
A CXR of a 41-year-old man with cirrhosis and PPH show enlarged hila and peripheral pruning of the vessels secondary to PH. An axial contrast enhanced CT through the pulmonary arteries of another patient with PPH showing aneurismal dilatation of the main pulmonary artery and the right pulmonary artery with peripheral pruning due to PH

**Figure 12:**
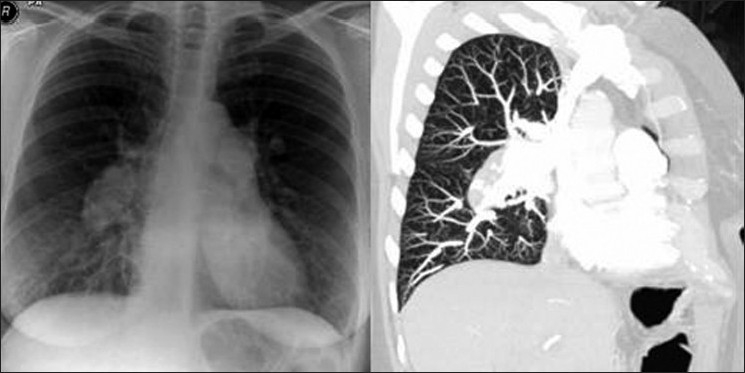
A CXR on a patient with PPH secondary to cirrhosis shows a dilated pulmonary conus and aneurismal dilatation of the right pulmonary artery. The image on the left is coronal reconstruction of contrast enhanced CT on the same patient, which depicts a probable re-canalized thrombus underlying the aneurismal dilatation of the right pulmonary artery

**Figure 13:**
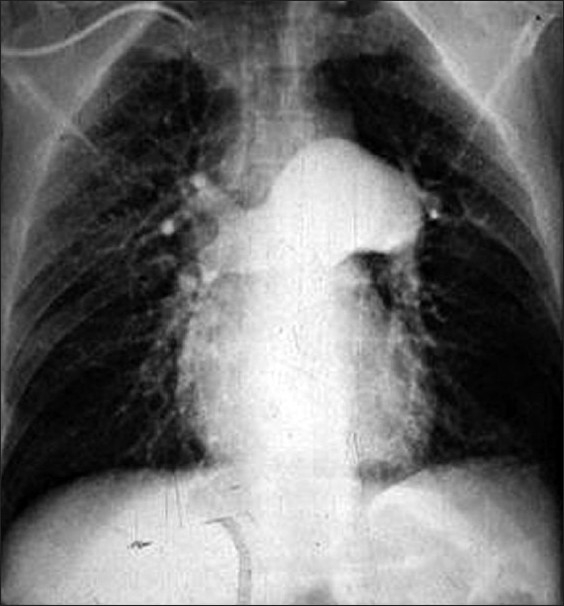
Pulmonary angiography on a patient with PPH showing dilatation of the pulmonary arteries and pruning of the peripheral vessels

A CXR may be normal in the early stages. As the disease advances the central pulmonary arteries become prominent with a right ventricular enlargement and tapering of the peripheral vessels. At CT, PH can be diagnosed when the diameter of the main pulmonary artery is greater than or equal to 29 mm accompanied by a segmental artery-to-bronchus ratio greater than 1:1 in three of four pulmonary lobes or/and the ratio of the main pulmonary artery diameter to the aortic diameter is greater than 1, particularly in patients less than 50 years of age[52 53] [Figures 11–13]. HRCT may show a mosaic pattern of lung attenuation is a nonspecific finding common to all types for PH but is relatively specific for thromboembolic PH. Radionuclide lung perfusion scans may be normal or show diffuse patchy perfusion defects in severe PH.

### Therapeutic measures

Optimal treatment for PPH remains elusive. Mild PPH with pulmonary artery pressure <35 mmHg is generally not treated. Moderate-to-severe PPH attempts are made to improve symptoms and lower pulmonary artery pressures and pulmonary vascular resistance. Anticoagulant therapy in PPH is generally not considered because of gastroesophageal varices and coagulation abnormalities related to liver disease, but anticoagulation should be considered in the absence of contraindications. Diuretics provide standard therapy in patients with PH and PPH, for the underlying liver disease, especially if ascites or peripheral edema is present. Oxygen therapy is used to treat hypoxemia. β-Blockers are used in some patients as prophylaxis of variceal bleeding. However, the use of β-Blockers in PPH is controversial as a significant worsening of exercise capacity has been reported after its use.[54]

A variety of vasodilators have been used in PPH. However, most of the studies of the use of vasodilator drugs have been used in PH from other causes. Prostanoids have been used successfully to lower pulmonary pressures in PPH. Epoprostenol has short half-life, a complicated delivery regime and is associated with several complications but has been shown to improve exercise capacity in PPH, although a survival advantage has not been documented to date.[55] Iloprost has similar effect and is used by inhalation but is very short-acting.[55] Bosentan is an endothelin receptor blocker, which is approved by FDA for the treatment of PH and PPH but is associated with mild hepatic dysfunction. However, irreversible hepatic toxicity is uncommon. Larger studies in the use of Bosentan in PPH are required. Ambrisentan has recently been approved for the treatment of PH. No studies are presently available for its use in PPH. Mild hepatic dysfunction may occur.[56] Sildenafil has been shown to increase cardiac output and decrease pulmonary artery pressures and pulmonary vascular resistance without serious adverse events in PH. Recent case series of 14 patients shows that sildenafil might be effective in PPH.[57]

OLT is not the treatment of choice for PPH, but after optimal hemodynamic and clinical improvement with medical therapy, OLT can be considered an option in selected patients.[58] PPH increases the risk of intraoperative and immediate postoperative complications of OLT. OLT should not be undertaken in patients with mean pulmonary artery pressures > 50 mm Hg whilst patients with mean pulmonary artery pressure between 35 and 50 mm Hg also have an increased mortality rate but may benefit from prolonged treatment for PH. Sildenafil use to treat PPH prior to OLT has met with some success

PPH is an uncommon complication of ESLD in children. Epoprostenol can bridge PPH patients to OLT. OLT leads to rapid resolution of HPS and PPH and currently represents the only successful treatment for these children.[59] Most successful OLT in PPH has been reported from unrelated cadaver donors.[60] One successful case of living-related liver transplantation in a patient with PPH has been published.[61]

## Conclusions

The major pulmonary vascular complications of CLD include HPS and PPH. The pathogenesis, imaging, treatment, and prognostic implications of these complications are discussed. In the context of ongoing uncertainty about causation and treatment of the HPS, future studies must focus on better understanding the pathogenesis of the HPS, predicting reversibility after OLT, and identifying other treatment options It is emphasized that appropriate knowledge of the pathogenesis of vascular complications and imaging of CLD would assist the clinician in initiating the appropriate treatment at the most appropriate time.
